# Supplementary material for: microRNA-33 maintains adaptive thermogenesis via enhanced sympathetic nerve activity
Source: Nat Commun. 2021 Feb 16;12:843. doi: 10.1038/s41467-021-21107-5 (PMC7886914; doi:10.1038/s41467-021-21107-5)
Supplement: Supplementary file 1 — Supplementary Information [file 41467_2021_21107_MOESM1_ESM.pdf]

Supplementary information

microRNA-33 maintains adaptive thermogenesis via enhanced sympathetic nerve activity

Takahiro Horie[1], Tetsushi Nakao[1], Yui Miyasaka[1], Tomohiro Nishino[1], Shigenobu Matsumura[2], Fumiko Nakazeki[1], Yuya Ide[1], Masahiro Kimura[1], Shuhei Tsuji[1], Randolph Ruiz Rodriguez[1], Toshimitsu Watanabe[1], Tomohiro Yamasaki[1], Sijia Xu[1], Chiharu Otani[1], Sawa Miyagawa[1], Kazuki Matsushita[1], Naoya Sowa[1], Aoi Omori[1], Jin Tanaka[2], Chika Nishimura[3], Masataka Nishiga[1], Yasuhide Kuwabara[1], Osamu Baba[1], Shin Watanabe[1], Hitoo Nishi[1], Yasuhiro Nakashima[1], Marina R. Picciotto[4], Haruhisa Inoue[5-7], Dai Watanabe[3], Kazuhiro Nakamura[8], Tsutomu Sasaki[9], Takeshi Kimura[1], and Koh Ono[1]

1. Department of Cardiovascular Medicine, Graduate School of Medicine, Kyoto University, Kyoto, Japan.
2. Laboratory of Physiological Functions of Food, Division of Food Science and Biotechnology, Graduate School of Agriculture, Kyoto University, Kyoto, Japan
3. Department of Biological Sciences, Graduate School of Medicine, Kyoto University, Kyoto, Japan
4. Department of Psychiatry and Interdepartmental Neuroscience Program, Yale University School of Medicine, New Haven, Connecticut
5. Center for iPS Cell Research and Application (CiRA), Kyoto University, Kyoto, Japan
6. iPSC-based Drug Discovery and Development Team, RIKEN BioResource Research Center (BRC), Kyoto, Japan
7. Medical-risk Avoidance based on iPS Cells Team, RIKEN Center for Advanced Intelligence Project (AIP), Kyoto, Japan
8. Department of Integrative Physiology, Nagoya University Graduate School of Medicine, Nagoya, Japan.
9. Laboratory of Nutrition Chemistry, Division of Food Science and Biotechnology, Graduate School of Agriculture, Kyoto University, Kyoto, Japan

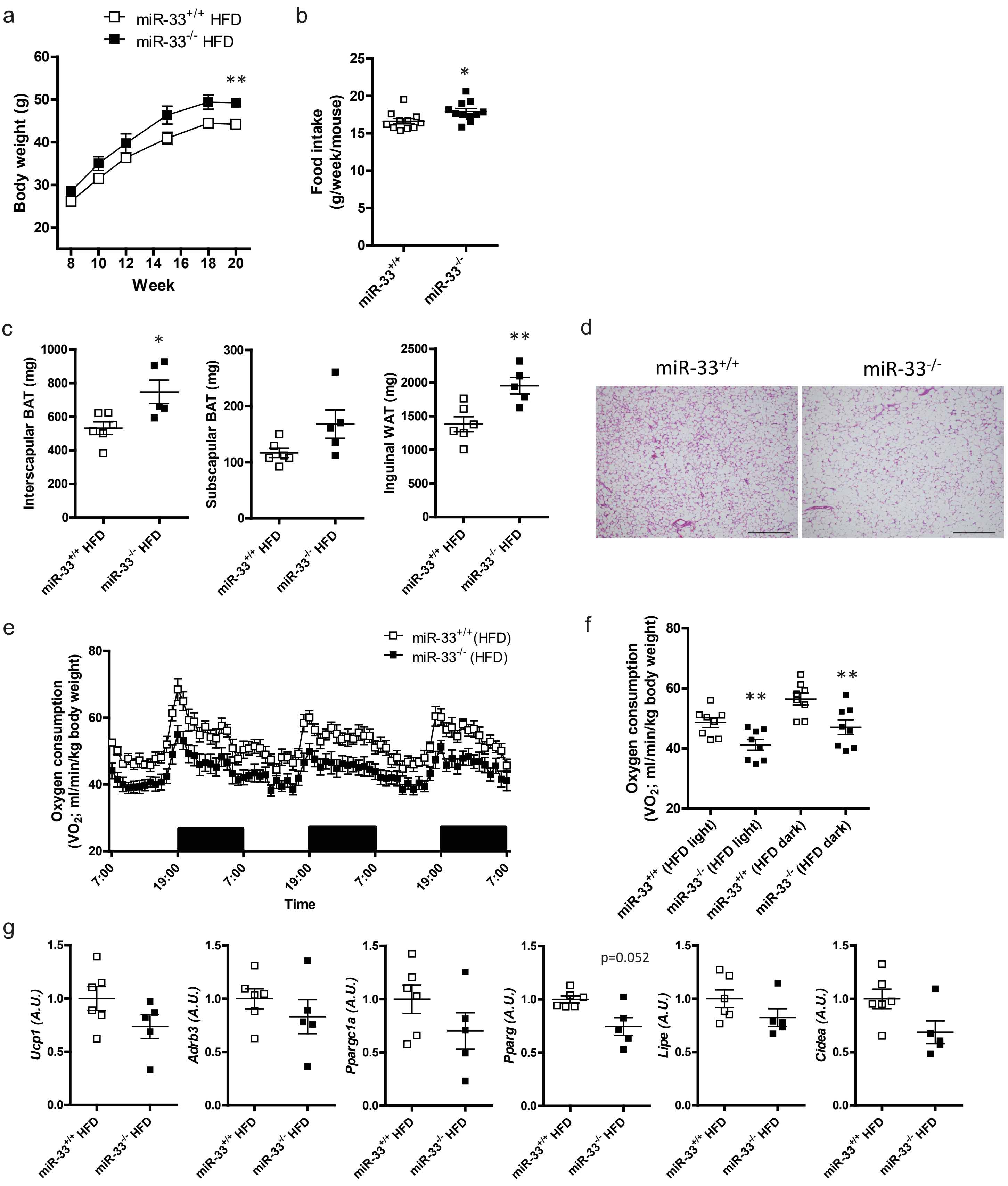

Supplementary Fig. 1. miR-33$^{-/-}$ mice become obese with reduced oxygen consumption with an HFD feeding. (a) Serial body weight change of miR-33$^{+/+}$ and miR-33$^{-/-}$ mice fed a 45% HFD feeding. n = 6, 5 mice per group, **p < 0.01, two-sided Mann–Whitney test. (b) Food intake of an HFD in miR-33$^{+/+}$ and miR-33$^{-/-}$ mice for 12 weeks. *p < 0.05, two-sided unpaired t-test. (c) Weight of interscapular BAT, subscapular BAT, and inguinal WAT of miR-33$^{+/+}$ and miR-33$^{-/-}$ mice fed a 45% HFD for 12 weeks. n = 6, 5 per group, **p < 0.01, *p < 0.05, two-sided unpaired t-test. (d) Representative images of HE staining for the BAT of miR-33$^{+/+}$ and miR-33$^{-/-}$ mice fed a 45% HFD for 12 weeks. n = 6, 5 mice per group. Scale bar, 200 μm. (e) Oxygen consumption rate in miR-33$^{+/+}$ and miR-33$^{-/-}$ mice fed a 45% HFD for 12 weeks. n = 8 mice per group, **p < 0.01, fdANOVA. (f) Mean oxygen consumption rate during the light and dark phase in miR-33$^{+/+}$ and miR-33$^{-/-}$ mice fed a 45% HFD for 12 weeks. n = 8 mice per group, **p < 0.01, two-sided unpaired t-test. (g) Quantitative real-time PCR analysis of thermogenic genes in the BAT of miR-33$^{+/+}$ and miR-33$^{-/-}$ mice fed a 45% HFD for 12 weeks. n = 6, 5 mice per group, two-sided Mann–Whitney test. All data are presented as mean ± SEM.

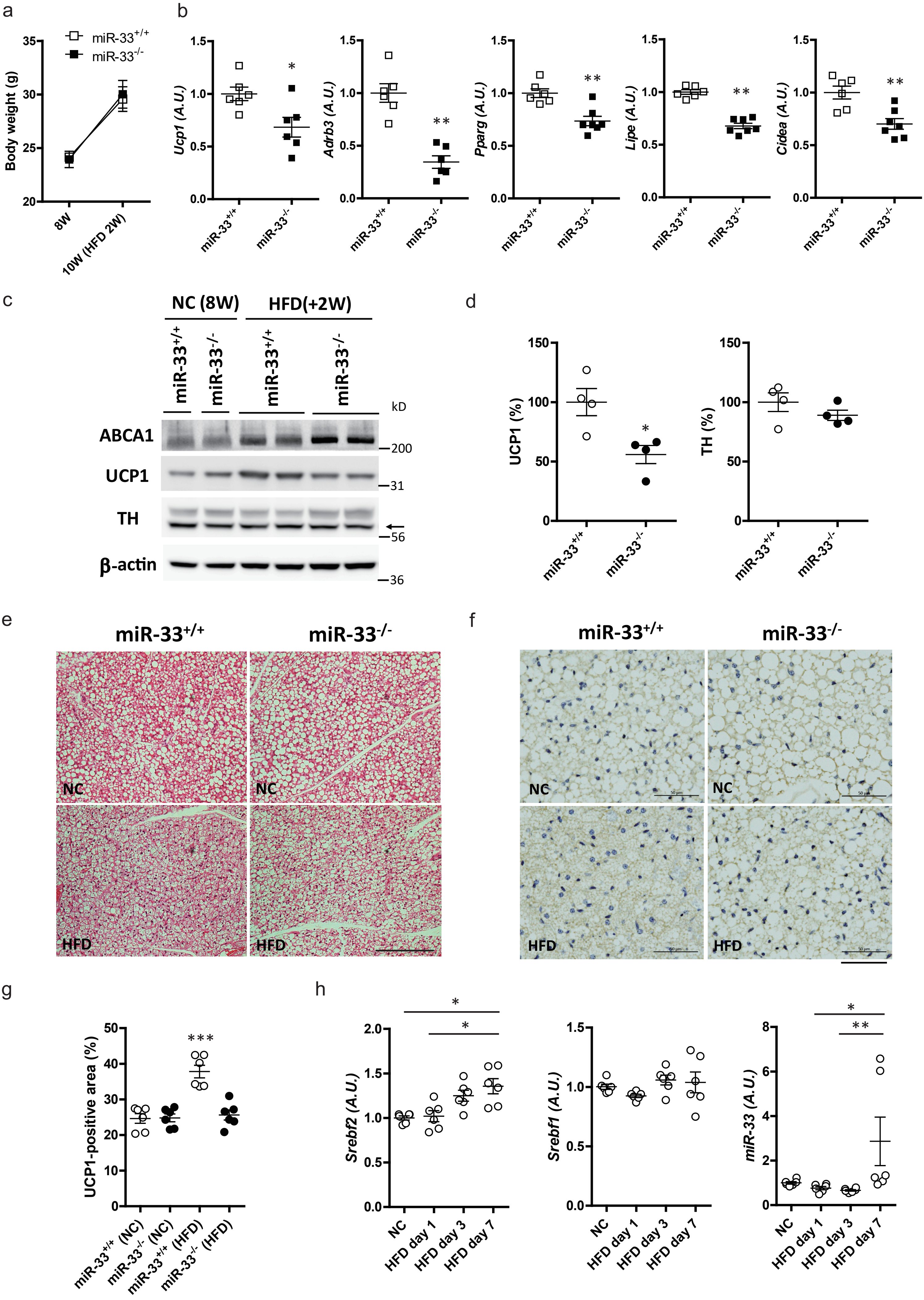

Supplementary Fig. 2. (Continued)

Supplementary Fig. 2. miR-33$^{-/-}$ mice show impaired thermogenic response with a short-term HFD feeding. (a) Body weight change of miR-33$^{+/+}$ and miR-33$^{-/-}$ mice fed a 60% HFD for 2 weeks. n = 6-7 mice per group. (b) Quantitative real-time PCR analysis of thermogenic genes in the BAT of miR-33$^{+/+}$ and miR-33$^{-/-}$ mice fed a 60% HFD for 2 weeks. n = 6-7 mice per group, **$p < 0.01$, *$p < 0.05$, two-sided Mann–Whitney test. (c) Western blotting analysis of ABCA1, UCP1, TH, and β-actin in the BAT of miR-33$^{+/+}$ and miR-33$^{-/-}$ mice fed normal chow (NC) and a 60% HFD for 2 weeks. The arrow indicates the specific bands of TH staining. (d) Densitometry for UCP1 and TH in the BAT of miR-33$^{+/+}$ and miR-33$^{-/-}$ mice fed a 60% HFD for 2 weeks. n = 4 per group, *$p < 0.05$, two-sided Mann-Whitney test. (e) Representative images of HE staining for the BAT of miR-33$^{+/+}$ and miR-33$^{-/-}$ mice fed normal chow (NC) and a 60% HFD for 2 weeks. n = 6 mice per group. Scale bar, 200 μm. (f) Representative images of UCP1 immunohistochemistry for the BAT of miR-33$^{+/+}$ and miR-33$^{-/-}$ mice fed NC and a 60% HFD for 2 weeks. n = 6 mice per group. Scale bar, 50 μm. (g) UCP1-positive area (%) in the BAT of miR-33$^{+/+}$ and miR-33$^{-/-}$ mice fed NC and a 60% HFD for 2 weeks. n = 6 mice per group. ***$p < 0.001$, compared with other groups, one-way ANOVA with Bonferroni's post hoc test. (h) Quantitative real-time PCR analysis of *Srebf2*, *Srebf1*, and miR-33 in the hypothalamus with a 60% HFD in wild-type mice. n = 6 mice per group, **$p < 0.01$, *$p < 0.05$, Kruskal-Wallis test with Dunn's post hoc test. All data are presented as mean ± SEM.

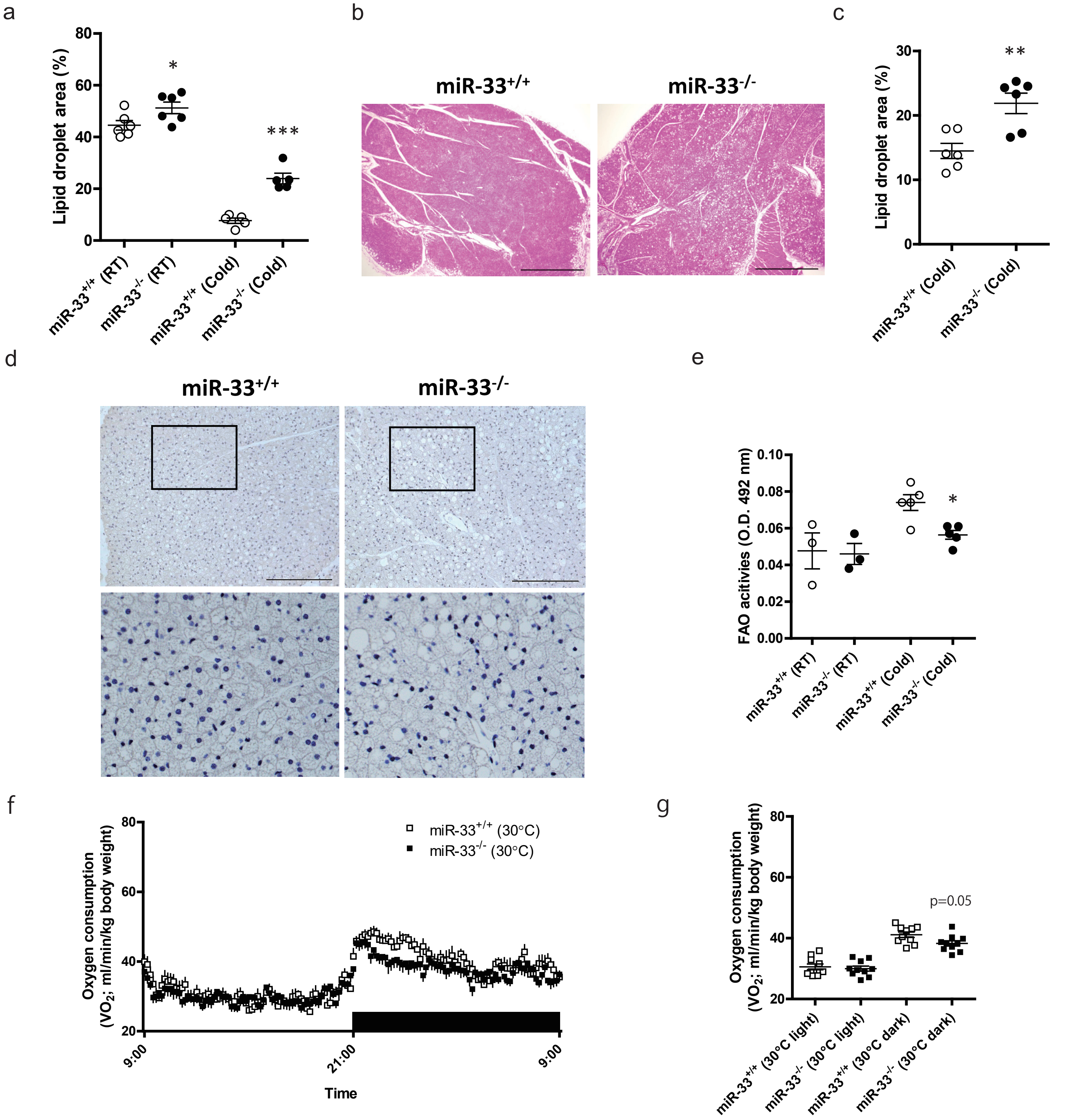

Supplementary Fig. 3. Reduced thermogenesis in miR-33$^{-/-}$ mice at a cold environment. (a) Quantification of lipid droplet area in the BAT of miR-33$^{+/+}$ and miR-33$^{-/-}$ mice kept at room temperature and at 4°C for 6 h. n = 6 mice per group for room temperature, n = 5 mice per group for cold exposure, ***$p < 0.001$, *$p < 0.05$, two-sided unpaired t-test. (b) Representative small-magnified images of HE staining for the BAT of miR-33$^{+/+}$ and miR-33$^{-/-}$ mice kept at 4°C for 48 h. n = 6 mice per group. Scale bar, 1 mm. (c) Quantification of lipid droplet area in the BAT of miR-33$^{+/+}$ and miR-33$^{-/-}$ mice kept at 4°C for 48 h. n = 6 mice per group, **$p < 0.01$, two-sided unpaired t-test. (d) Representative images of immunohistochemistry for UCP1 in the BAT of miR-33$^{+/+}$ and miR-33$^{-/-}$ mice kept at 4°C for 48 h. n = 6 mice per group. Scale bar, 200 μm. (e) Fatty acid oxidation activity in the BAT of miR-33$^{+/+}$ and miR-33$^{-/-}$ mice at room temperature and two days after transfer from 23°C to 18°C. n = 3 for room temperature, n = 5 for cold exposure, *$p < 0.05$, two-sided Mann–Whitney test. (f) Oxygen consumption rate of miR-33$^{+/+}$ and miR-33$^{-/-}$ mice kept at 30°C. n = 10 mice per group. (g) Mean oxygen consumption rate during the light and dark phase in miR-33$^{+/+}$ and miR-33$^{-/-}$ mice kept at 30°C. n = 10 mice per group, two-sided Mann-Whitney test. All data are presented as mean ± SEM.

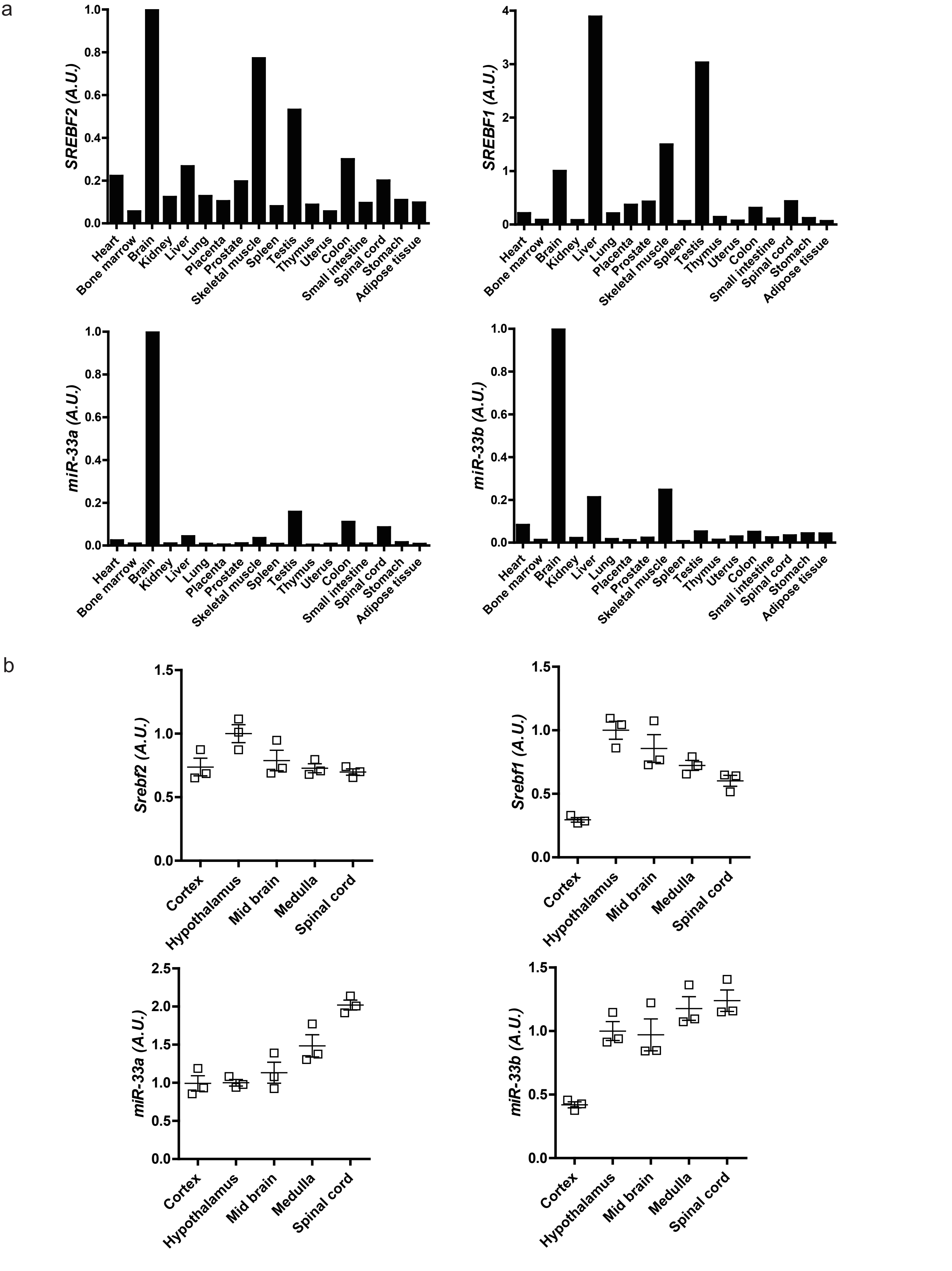

Supplementary Fig. 4. Expression of miR-33a and miR-33b in human organs and the brain of miR-33b$^{+/+}$ mice. (a) Quantitative real-time PCR analysis of *SREBF2*, *SREBF1*, miR-33a, and miR-33b in human organs. n = 1 per group. The expression of brain is set to 1. (b) Quantitative real-time PCR analysis of *Srebf2*, *Srebf1*, miR-33a, and miR-33b in several regions of the central nervous system in miR-33b$^{+/+}$ mice. n = 3 per group. The expression of hypothalamus is set to 1. All data are presented as mean ± SEM.

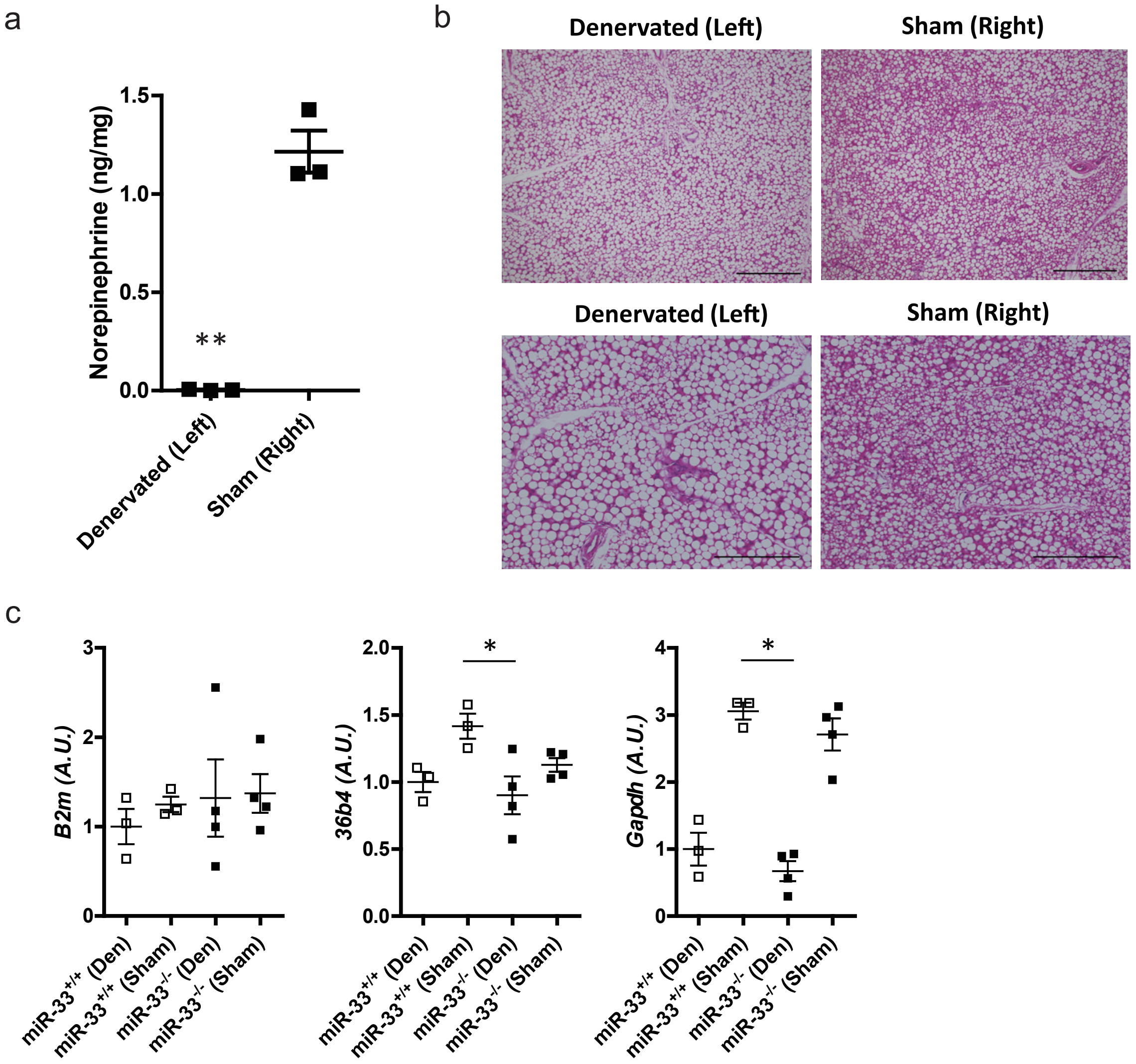

Supplementary Fig. 5. Assessment of sympathetic nerve activity of the BAT by denervation of sympathetic nerves. (a) Norepinephrine contents in the denervated and sham-operated BAT in wild-type mice. The left-side BAT was denervated and the right-side BAT was sham-operated. At 1 week after operation, mice were analyzed. n = 3 mice per group, **p < 0.01, two-sided paired t-test. (b) Representative images of HE staining of the denervated and sham-operated BAT in wild-type mice. n = 3 mice per group. Scale bar, 300 μm (upper), 200 μm (lower). (c) Quantitative real-time PCR analysis of *B2m*, *36b4*, and *Gapdh* in the denervated or sham-operated BAT of miR-$33^{+/+}$ and miR-$33^{-/-}$ mice kept at 4°C for 5 h. At 1 week after operation, mice were transferred to 4°C for 5 h. n = 3, 4 mice per group. *p < 0.05, Kruskal-Wallis test with Dunn's post hoc test. All data are presented as mean ± SEM.

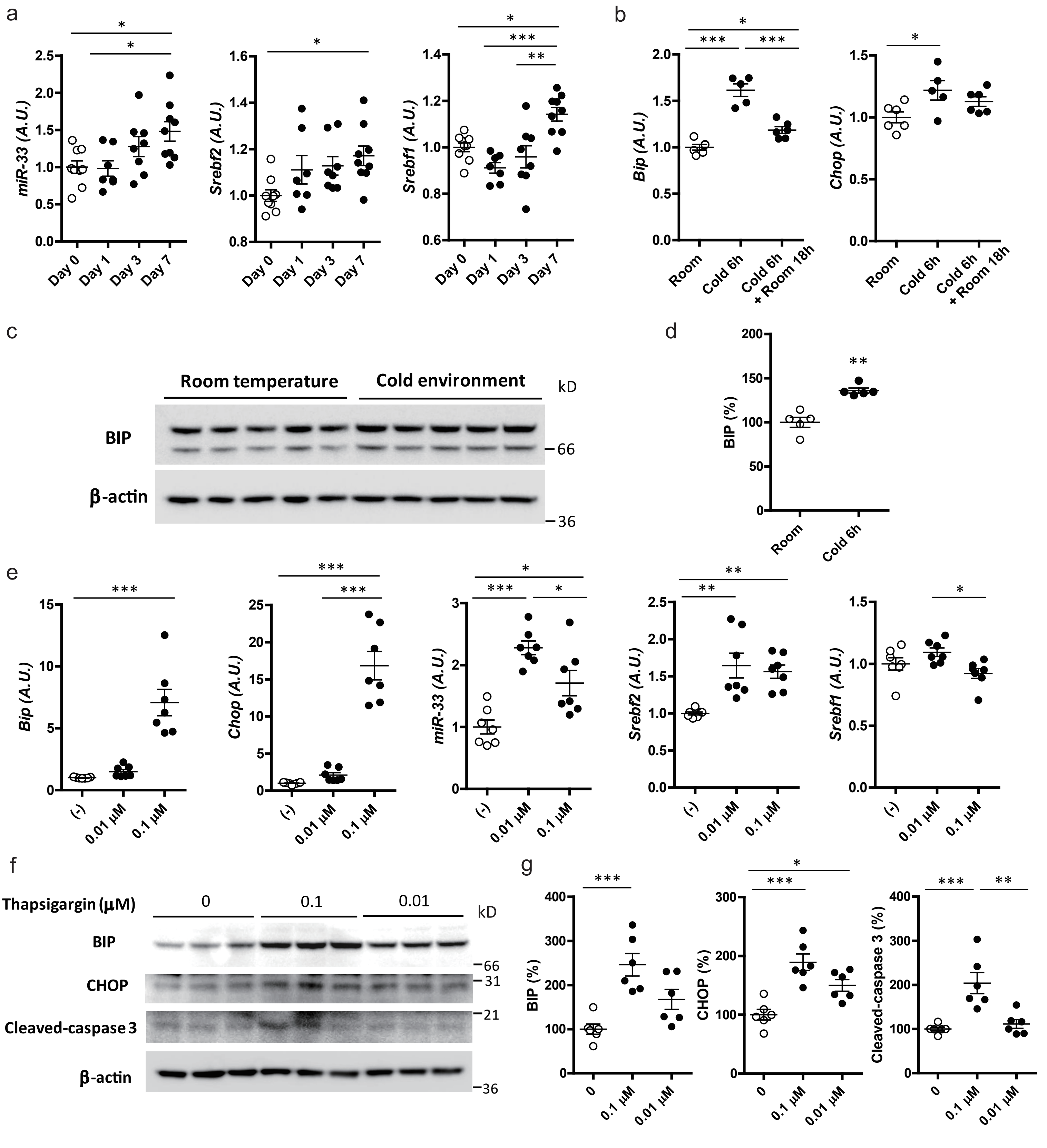

Supplementary Fig. 6. (Continued)

Supplementary Fig. 6. Cold exposure induces hypothalamic miR-33 possibly via ER stress. (a) Quantitative real-time PCR analysis of miR-33, *Srebf2*, and *Srebf1* in the hypothalamus with intermittent cold exposure in wild-type mice. n = 9 for Day 0, n = 7 for Day 1, n = 8 for Day 3, n = 9 for Day 7, ***$p < 0.001$, **$p < 0.01$, *$p < 0.05$, one-way ANOVA with Bonferroni's post hoc test. (b) Quantitative real-time PCR analysis of *Bip* and *Chop* in the hypothalamus with cold exposure for 6 h and at additional 18 h at room temperature in wild-type mice. n = 6 for room temperature, n = 5 for 6 h cold exposure, n = 6 for 6 h cold exposure and 18 h room temperature, ***$p < 0.001$, *$p < 0.05$, one-way ANOVA with Bonferroni's post hoc test. (c) Western blotting for BIP and β-actin in the hypothalamus at room temperature or with cold exposure for 6 h in wild-type mice. n = 5 mice per group. (d) Densitometric analysis for BIP in the hypothalamus at room temperature or with cold exposure for 6 h in wild-type mice. n = 5 mice per group. **$p < 0.01$, two-sided Mann-Whitney test. (e) Quantitative real-time PCR analysis of *Bip*, *Chop*, miR-33, *Srebf2*, and *Srebf1* in Neuro2a cells under thapsigargin treatment for 2 h with the indicated concentrations. Cells were harvested at 24 h after treatment. n = 7 per each, ***$p < 0.001$, **$p < 0.01$, *$p < 0.05$, one-way ANOVA with Bonferroni's post hoc test. (f) Western blotting for BIP, CHOP, cleaved-caspase 3, and β-actin in Neuro2a cells under thapsigargin treatment for 2 h with the indicated concentrations. Cells were harvested at 24 h after treatment. n = 3 per each, Representative images from 2 independent experiments. (g) Densitometric analysis for BIP, CHOP and cleaved-caspase 3 in in Neuro2a cells under thapsigargin treatment for 2 h with the indicated concentrations. Cells were harvested at 24 h after treatment. n = 6 per each, ***$p < 0.001$, *$p < 0.05$, one-way ANOVA with Bonferroni's post hoc test. All data are presented as mean ± SEM.

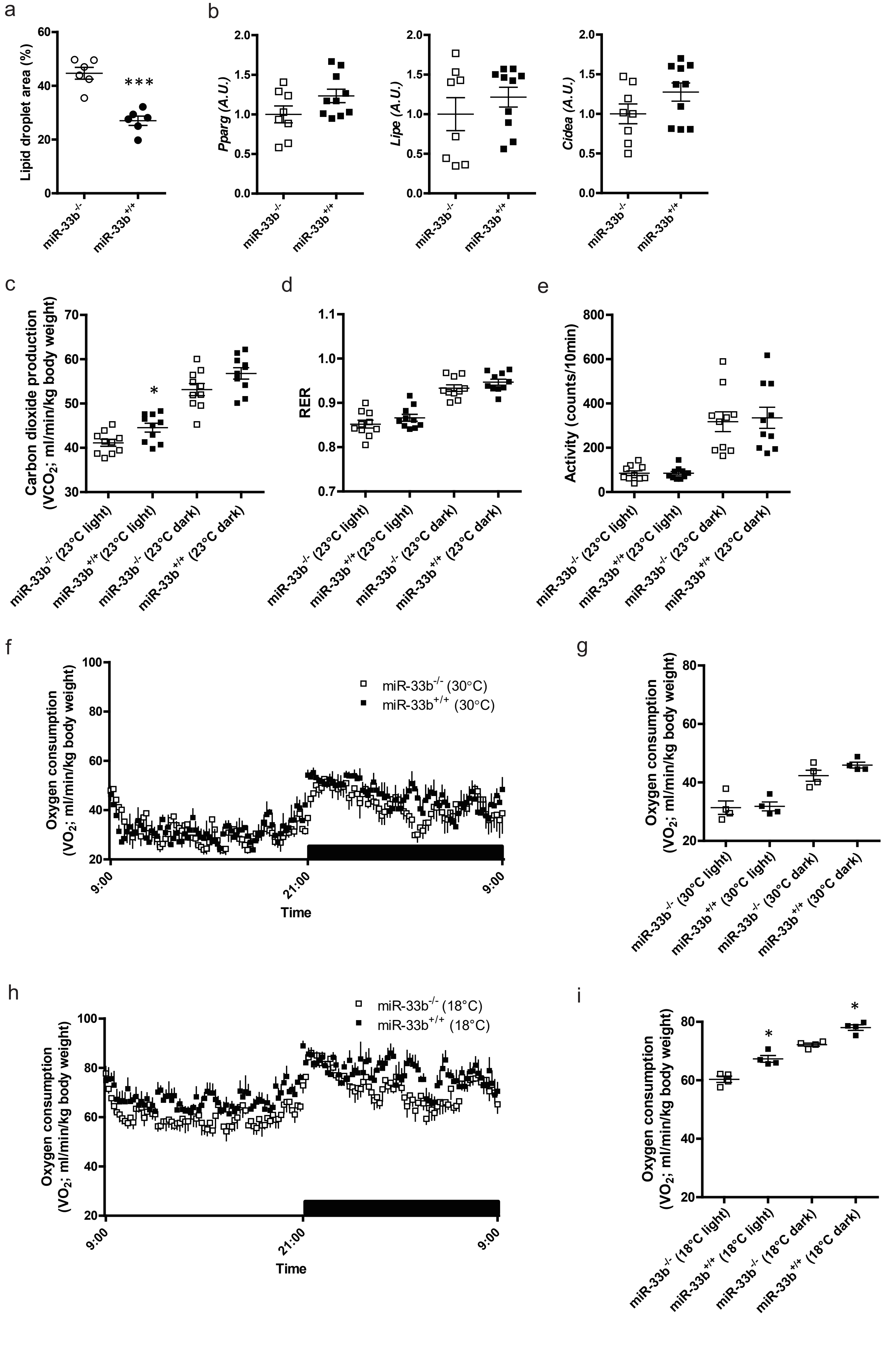

Supplementary Fig. 7. (Continued)

j

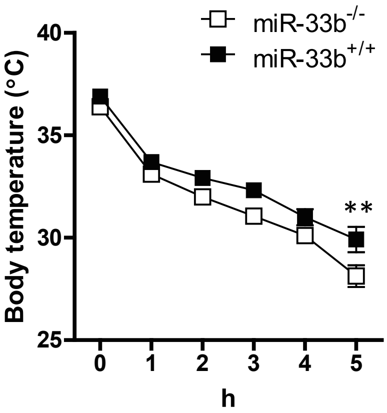

k

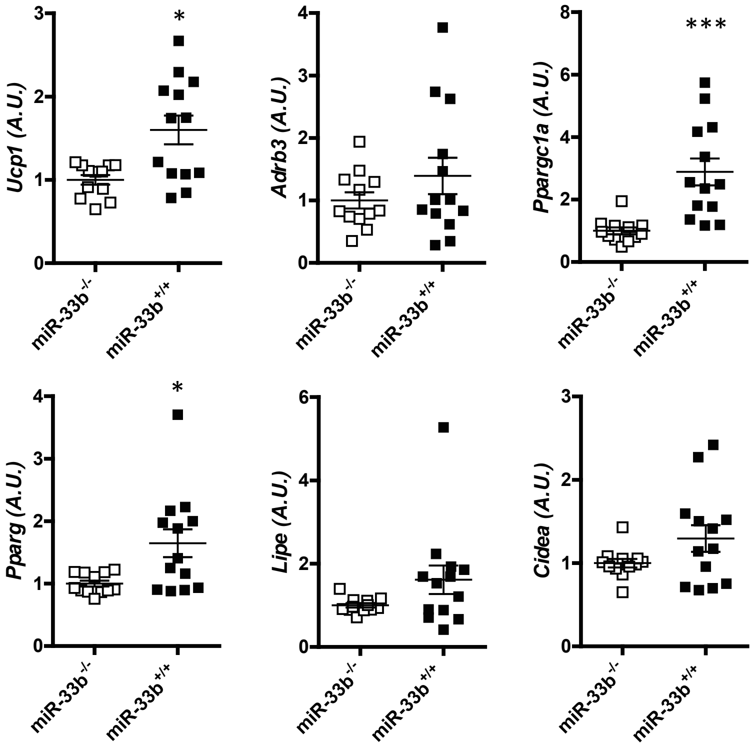

l

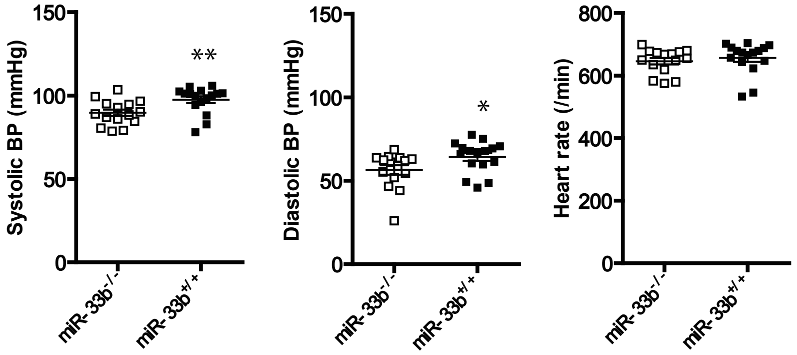

Supplementary Fig. 7. miR-33b$^{+/+}$ mice exhibit increased BAT activity. (a) Quantification of lipid droplet area in the BAT of miR-33b$^{-/-}$ and miR-33b$^{+/+}$ mice at room temperature. n = 6 mice per group, ***p < 0.001, two-sided unpaired t-test. (b) Quantitative real-time PCR analysis of *Pparg*, *Lipe*, and *Cidea* in the BAT of miR-33b$^{-/-}$ and miR-33b$^{+/+}$ mice at room temperature. n = 8, 10 mice per group. (c) Carbon dioxide production rate in miR-33b$^{-/-}$ and miR-33b$^{+/+}$ mice kept at 23°C. n = 10 mice per group. * p < 0.05, two-sided unpaired t-test. (d) Respiratory exchange rate in miR-33b$^{-/-}$ and miR-33b$^{+/+}$ mice kept at 23°C. n = 10 mice per group. (e) Locomotor activity of miR-33b$^{-/-}$ and miR-33b$^{+/+}$ mice kept at 23°C. n = 10 mice per group. (f) Oxygen consumption rate of miR-33b$^{-/-}$ and miR-33b$^{+/+}$ mice kept at 30°C. n = 4 mice per group. (g) Mean oxygen consumption rate during the light and dark phase in miR-33b$^{-/-}$ and miR-33b$^{+/+}$ mice at 30°C. n = 4 mice per group. (h) Oxygen consumption rate of miR-33b$^{-/-}$ and miR-33b$^{+/+}$ mice kept at 18°C n = 4 mice per group, *p < 0.05, fdANOVA. (i) Mean oxygen consumption rate during the light and dark phase in miR-33b$^{-/-}$ and miR-33b$^{+/+}$ mice at 18°C. n = 4 mice per group, *p < 0.05, two-sided Mann–Whitney test. (j) Serial core body temperature change of miR-33b$^{-/-}$ and miR-33b$^{+/+}$ mice kept at 4°C. n = 12, 13 mice per group, ** p < 0.01, two-way repeated measures ANOVA with Bonferroni's post hoc test. (k) Quantitative real-time PCR analysis of thermogenic genes in the BAT of miR-33b$^{-/-}$ and miR-33b$^{+/+}$ mice kept at 4°C. n = 12, 13 mice per group, ***p < 0.001, *p < 0.05, two-sided Mann–Whitney test. (l) Measurement of blood pressure and heart rate in miR-33b$^{-/-}$ and miR-33b$^{+/+}$ mice at room temperature. n = 15, 16 mice per group, **p < 0.01, *p < 0.05, two-sided Mann–Whitney test. All data are presented as mean ± SEM.

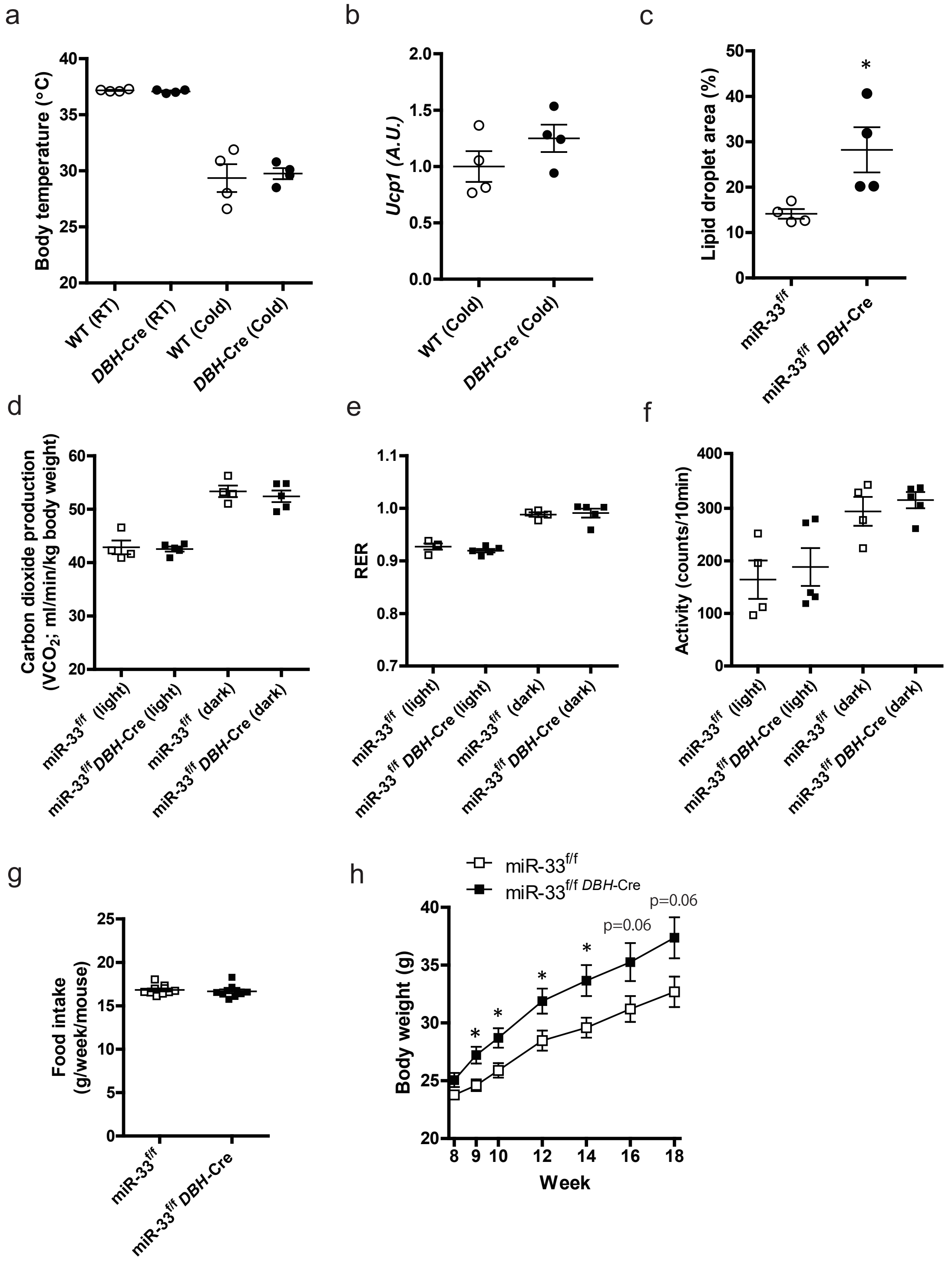

Supplementary Fig. 8. Generation of miR-33$^{f/f}$ *DBH*-Cre mice. (a) Core body temperature at room temperature and a cold environment of wild-type (WT) and *DBH*-Cre mice. n = 4 mice per group. (b) The expression of *Ucp1* in the BAT of wild-type (WT) and *DBH*-Cre mice at a cold environment. n = 4 mice per group. (c) Quantification of lipid droplet area in the BAT of miR-33$^{f/f}$ and miR-33$^{f/f}$ *DBH*-Cre mice kept at 4°C for 6 h. n = 4 mice per group, *p < 0.05, two-sided Mann–Whitney test. (d) Mean carbon dioxide production rate in miR-33$^{f/f}$ and miR-33$^{f/f}$ *DBH*-Cre mice kept at 18°C. n = 4, 5 mice per group. (e) Respiratory exchange rate of miR-33$^{f/f}$ and miR-33$^{f/f}$ *DBH*-Cre mice kept at 18°C. n = 4, 5 mice per group. (f) Locomotor activity of miR-33$^{f/f}$ and miR-33$^{f/f}$ *DBH*-Cre mice kept at 18°C. n = 4, 5 mice per group. (g) Food intake of an HFD in miR-33$^{f/f}$ and miR-33$^{f/f}$ *DBH*-Cre mice for 10 weeks. (h) Serial body weight change of miR-33$^{f/f}$ and miR-33$^{f/f}$ *DBH*-Cre fed a 45% HFD feeding. n = 7 mice per group, *p < 0.05, two-sided unpaired t-test. All data are presented as mean ± SEM.

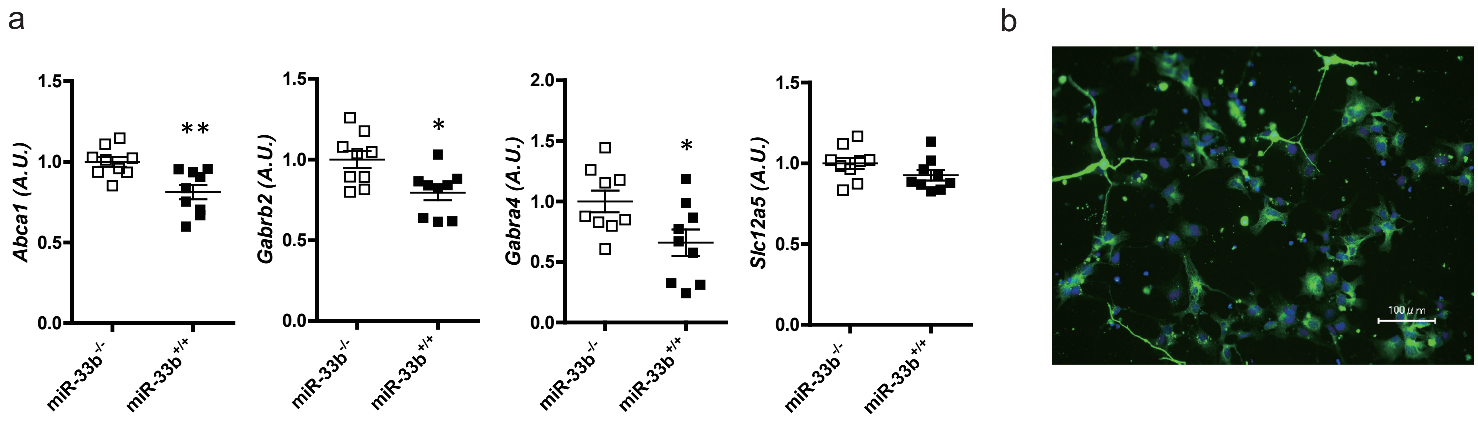

Supplementary Fig. 9. GABA-related gene expressions in miR-33b$^{+/+}$ hypothalamus. (a) Quantitative real-time PCR analysis of *Abca1*, *Gabrb2*, *Gabra4*, and *Slc12a5* in the hypothalamus of miR-33b$^{-/-}$ and miR-33b$^{+/+}$ mice. n = 9 mice per group, **p < 0.01, *p < 0.05, two-sided unpaired t-test. (b) Fluorescence image of human iPS cell-derived cortical neurons stained with β3-tubulin (green) and DAPI (blue). A representative image from two independent experiments. Scale bar, 100 μm. All data are presented as mean ± SEM.

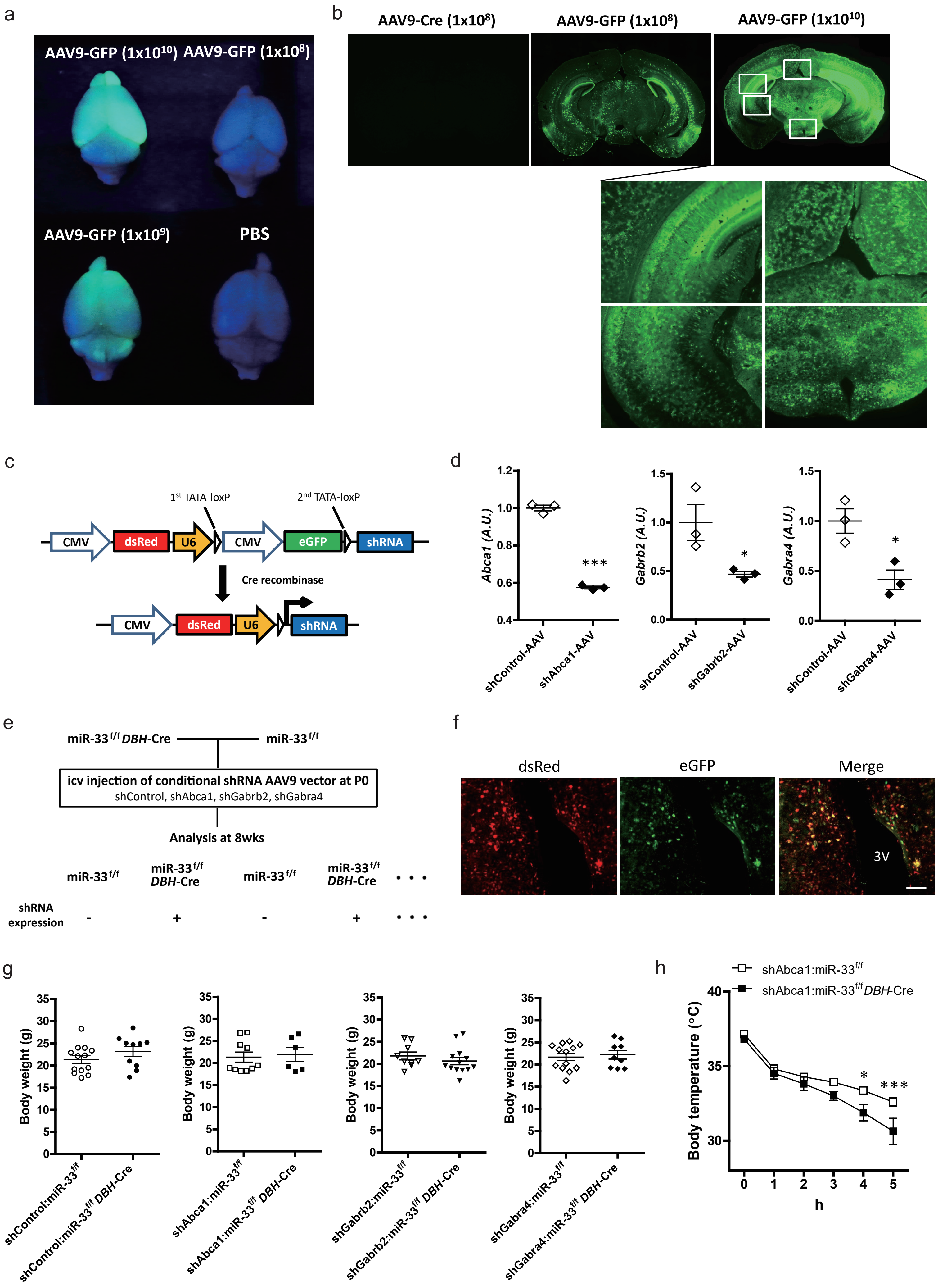

Supplementary Fig. 10. (Continued)

i

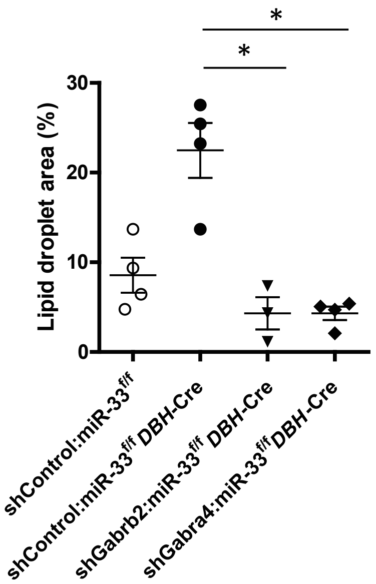

Supplementary Fig. 10. AAV9-mediated *in vivo* rescue experiment. (a) Fluorescent whole brain images with icv injection of AAV9-GFP vector. AAV9-GFP vectors of indicated particles were injected into wild-type neonatal (P0) brain by icv injection, and then mice were analyzed at the age of 8 weeks old. Representative images from two independent experiments. (b) Fluorescence images of brain slice with icv injection of AAV9-Cre or AAV9-GFP vector. Magnified images are shown of brains injected with AAV9-GFP vector at a dose of 10^{10} viral particles. Representative images from two independent experiments. (c) Scheme of the conditional shRNA AAV9 vector. shRNA is expressed under the control of Cre recombinase. (d) Validation of the efficiency of shRNA sequences used *in vivo* rescue experiment in Neuro2a cells. n = 3, ***p < 0.001, *p < 0.05, two-sided unpaired t-test. (e) Scheme of AAV9-mediated *in vivo* rescue experiment. Conditional shRNA AAV9 vectors were injected into the pops between miR-33$^{f/f}$ *DBH*-Cre and miR-33$^{f/f}$ mice at neonatal period (P0) by icv injection, and then mice were analyzed at the age of 8 weeks old. shRNAs were expressed only in *DBH*-positive cells in the brain of miR-33$^{f/f}$ *DBH*-Cre mice. (f) Representative fluorescence images of the region around the 3rd ventricle of miR-33$^{f/f}$ *DBH*-Cre mice brains infected with conditional control shRNA AAV9 vector. Representative images from two independent experiments. Scale bar, 100 µm. (g) Body weight of miR-33$^{f/f}$ and miR-33$^{f/f}$ *DBH*-Cre mice infected with conditional shRNA AAV9 vectors for control or against *Abca1*, *Gabrb2* and *Gabra4* at the age of 8 weeks. n = 13, 10 mice per group for shControl, n = 10, 6 mice per group for shAbca1, n = 9, 13 mice per h group for shGabrb2, n = 13, 9 mice per group for shGabra4. (h) Serial core body temperature changes in miR-33$^{f/f}$ and miR-33$^{f/f}$ *DBH*-Cre mice infected with conditional shRNA AAV9 vector against *Abca1* with 4°C. n = 10, 6 mice per group, *p < 0.05, *** p < 0.001, two-way repeated measures ANOVA with Bonferroni's post hoc test. (i) Quantification of lipid droplet area in the BAT of indicated mice kept at 4°C for 5 h, n = 4, 4, 3, 4 mice per group, *p < 0.05, Kruskal-Wallis test with Dunn's post hoc test. All data are presented as mean ± SEM.

Supplemental table 1. Primers for genotyping and product size

Primer	Sequence	Product (bp)
miR-33 WT and KO (F)	GGCACTACTTCTGATCCTTC	WT/KO 385/491
miR-33 WT (R)	CAACTACAATGCACCACAGCTG	
miR-33 KO (R)	TTGGGATCCAGAATTCGTGATTAA	
miR-33b (F)	ATGGATTTACCTCAGTTTTAACGAC	WT/KI 199/491
miR-33b (R)	AAGTGGATCCAGAATTCGTGA	
miR-33^{flox} (F)	TCTTCTCCCGAGCTCTCTTACTCTCA	floxed/WT/Δ 465/327/223
miR-33^{flox} (R)	TTATTCCCCACGATGGTTAGGTAGGC	
Cre (F)	GAACCTGATGGACATGTTCAGG	
Cre (R)	AGTGCGTTCGAACGCTAGAGCCTGT	320
Myogenin (internal control) (F)	TTACGTCCATCGTGGACAGC	
Myogenin (internal control) (R)	TGGGCTGGGTGTTAGCCTTA	250

Supplemental table 2. Primers used in quantitative real-time PCR in this study

Species	Gene	Sequence (F)	Sequence (R)
Mouse	*Ucp1*	TGTCCATGTACACCAAGGAAGG	TGTCTGTCTGGACTTCATCAGC
	Adrb3	TATGGGCATCTTCTCTCTGTGC	CAGTTCAGGGCGATGAAAACTC
	Ppargc1a	ATCACGTTCAAGGTCACCCTAC	GCTTCTGCCTCTCTCTCTGTTT
	Pparg	CCCACCAACTTCGGAATCAG	TGCTGGAGAAATCAACTGTGGTA
	Lipe	ACTCAGACCAGAAGGCACTA	TAGTTCCAGGAAGGAGTTGA
	Cidea	ATGATCTTGGAAAAGGGACAGA	GCCTGTATAGGTCGAAGGTGAC
	B2m	TGCTATCCAGAAAACCCCTCA	GCGGGTGGAACTGTGTTACG
	36b4	CAACCCAGCTCTGGAGAAAC	CCAACAGCATATCCCGAATC
	Gapdh	AAATGGTGAAGGTCGGTGTG	AATCTCCACTTTGCCACTGC
	Elovl3	ACCTACATGAGAACGCGGAA	TGTAGATGGCAAAGCACACG
	Abca1	AACAGTTTGTGGCCCTTTTG	AGTTCCAGGCTGGGGTACTT
	Gabrb2	GCTGCTAATGCCAACAATGA	CCCATTACTGCTTCGGATGT
	Gabra4	TAAACGAATCCCCAGGACAG	GACGCAGCCTGTTGTCATAA
	Slc12a5	TTTGCTGCTCCTGTACGATG	GTGCCCAGGTAGAAGCAGAG
	Srebf1	TAGAGCATATCCCCCAGGTG	GGTACGGGCCACAAGAAGTA
	Srebf2	GTGGAGCAGTCTCAACGTCA	TGGTAGGTCTCACCCAGGAG
	Bip	CTGAGGCGTATTTGGGAAAG	TCATGACATTCAGTCCAGCAA
	Chop	AGCTGGAAGCCTGGTATGAGGA	AGCTAGGGACGCAGGGTCAA
	Actb	GATCTGGCACCACACCTTCT	GGGGTGTTGAAGGTCTCAAA
Human	*SREBF1*	AACAGTCCCACTGGTCGTAGAT	TGTTGCAGAAAGCGAATGTAGT
	SREBF2	AGGAGAACATGGTGCTGA	TAAAGGAGAGGCACAGGA
	GABRB2	CTGGACAACAGAGTGGCAGA	ATAAAGGACGGTGCCATCAG
	GABRA4	AGGGGCTACTGGGAAGTTGT	ACCATGTTAAATGCCCCAAA
	SLC12A5	TGCTCCTGTACGATGCTCAC	GTGCCCAGGTAGAAGCAGAG
	ACTB	AGGCACTCTTCCAGCCTTCC	GCACTGTGTTGGCGTACAGG

Supplementary Table 3. Sequences for shRNA used *in vivo* rescue study

shRNA	Sequence
shRNA *Control*	AAATGTACTGCGCGTGGAGAC
shRNA *Abca1*	GAAGAATCTGACATTTCGAAG
shRNA *Gabrb2*	GCAGCTGAGAAAGCTGCTAAT
shRNA *Gabra4*	GCAAATGCAGCTGAGACTATG
