# Supplementary material for: microRNA-33 maintains adaptive thermogenesis via enhanced sympathetic nerve activity
Source: Nat Commun. 2021 Feb 16;12:843. doi: 10.1038/s41467-021-21107-5 (PMC7886914; doi:10.1038/s41467-021-21107-5)
Supplement: Supplementary file 3 — Reporting Summary [file 41467_2021_21107_MOESM3_ESM.pdf]

nature research

Corresponding author(s): Koh Ono

Last updated by author(s): Nov 28, 2020

# Reporting Summary

Nature Research wishes to improve the reproducibility of the work that we publish. This form provides structure for consistency and transparency in reporting. For further information on Nature Research policies, see our Editorial Policies and the Editorial Policy Checklist.

## Statistics

For all statistical analyses, confirm that the following items are present in the figure legend, table legend, main text, or Methods section.

| n/a | Confirmed | |
|---|---|---|
| ☐ | ☒ | The exact sample size (*n*) for each experimental group/condition, given as a discrete number and unit of measurement |
| ☐ | ☒ | A statement on whether measurements were taken from distinct samples or whether the same sample was measured repeatedly |
| ☐ | ☒ | The statistical test(s) used AND whether they are one- or two-sided<br>*Only common tests should be described solely by name; describe more complex techniques in the Methods section.* |
| ☐ | ☒ | A description of all covariates tested |
| ☐ | ☒ | A description of any assumptions or corrections, such as tests of normality and adjustment for multiple comparisons |
| ☐ | ☒ | A full description of the statistical parameters including central tendency (e.g. means) or other basic estimates (e.g. regression coefficient) AND variation (e.g. standard deviation) or associated estimates of uncertainty (e.g. confidence intervals) |
| ☐ | ☒ | For null hypothesis testing, the test statistic (e.g. *F*, *t*, *r*) with confidence intervals, effect sizes, degrees of freedom and *P* value noted<br>*Give P values as exact values whenever suitable.* |
| ☒ | ☐ | For Bayesian analysis, information on the choice of priors and Markov chain Monte Carlo settings |
| ☒ | ☐ | For hierarchical and complex designs, identification of the appropriate level for tests and full reporting of outcomes |
| ☒ | ☐ | Estimates of effect sizes (e.g. Cohen's *d*, Pearson's *r*), indicating how they were calculated |

*Our web collection on statistics for biologists contains articles on many of the points above.*

## Software and code

Policy information about availability of computer code

| | |
|---|---|
| Data collection | Quantitative real time PCR: StepOnePlus real-time PCR system and StepOne Software v2.3<br>Western blotting: ImageQuant LAS 4000 mini and ImageJ 1.44p<br>Metabolic rate: Oxymax and CLAX software v2.2.10<br>Image: Axio Observer 7 and Zen 2 pro software<br>Sequencing: 3130xl Genetic Analyzer, Foundation Data Collection v3.1.1 and Sequence Analysis Software v5.4 |
| Data analysis | GraphPad Prism 5.04 and 6.05, ImageJ 1.44p, R software 3.6.0 |

For manuscripts utilizing custom algorithms or software that are central to the research but not yet described in published literature, software must be made available to editors and reviewers. We strongly encourage code deposition in a community repository (e.g. GitHub). See the Nature Research guidelines for submitting code & software for further information.

## Data

Policy information about availability of data

All manuscripts must include a data availability statement. This statement should provide the following information, where applicable:

- Accession codes, unique identifiers, or web links for publicly available datasets
- A list of figures that have associated raw data
- A description of any restrictions on data availability

The data that support the findings in this study are available from the corresponding authors upon reasonable request.
Source data for figures are provided with the paper.

# Field-specific reporting

Please select the one below that is the best fit for your research. If you are not sure, read the appropriate sections before making your selection.

- [x] Life sciences
- [ ] Behavioural & social sciences
- [ ] Ecological, evolutionary & environmental sciences

For a reference copy of the document with all sections, see nature.com/documents/nr-reporting-summary-flat.pdf

# Life sciences study design

All studies must disclose on these points even when the disclosure is negative.

| | |
|---|---|
| Sample size | Calculation of sample size was not performed in this study. Sample size was determined based on previous studies and literatures or our pilot studies, whose numbers were described in the figure legends (Cell Metabolism 2014;20:41-53, 2012;16:823-32, Nat Commun. 2013;4;2883). |
| Data exclusions | No data were excluded, except for a rare case with failure of measurement due to technical problem such as poor amplification in quantitative RT-PCR. We excluded one data in Ppargc1a/Pparg in F1d, Adrb3 in F5g and Ucp1/Adrb3 in S2b. |
| Replication | The experiments were repeated at least twice and the reproducibility was confirmed. Quantitative RT-PCR and western blotting data were confirmed by three or more independent samples. |
| Randomization | Animals were randomized into the experimental groups with equivalent body weight. For cell experiments, randomization was not relevant, because cells were prepared and treated in the same way by the same researchers regardless of the treatments. |
| Blinding | The investigators were not blinded to group allocation during data collection and/or analysis, because mice needed to be genotyped by PCR and cells were treated by the researchers who collected the data. |

# Reporting for specific materials, systems and methods

We require information from authors about some types of materials, experimental systems and methods used in many studies. Here, indicate whether each material, system or method listed is relevant to your study. If you are not sure if a list item applies to your research, read the appropriate section before selecting a response.

## Materials & experimental systems

| n/a | Involved in the study | |
|---|---|---|
| [ ] | [x] | Antibodies |
| [ ] | [x] | Eukaryotic cell lines |
| [x] | [ ] | Palaeontology and archaeology |
| [ ] | [x] | Animals and other organisms |
| [x] | [ ] | Human research participants |
| [x] | [ ] | Clinical data |
| [x] | [ ] | Dual use research of concern |

## Methods

| n/a | Involved in the study | |
|---|---|---|
| [x] | [ ] | ChIP-seq |
| [x] | [ ] | Flow cytometry |
| [x] | [ ] | MRI-based neuroimaging |

## Antibodies

Antibodies used

anti-ABCA1 antibody, WB (1:1000), NB400-105; Novus Biologicals, CO
anti-UCP1 antibody, WB (1:2000) IHC (1:500), U6382, Sigma-Aldrich, MO
anti-β-actin antibody, WB (1:3000), A5441, Sigma-Aldrich, MO
anti-tyrosine hydroxylase antibody, WB (1:1000), IHC (1:250), AB152, Millipore, MA
anti-c-fos antibody, IHC (1:200), sc-52, Santa Cruz Biotechnology, CA
anti-PGC1a antibody, WB (1:500), H-300, Santa Cruz Biotechnology, CA
anti-CHOP antibody, WB (1:500), sc-575, Santa Cruz Biotechnology, CA
anti-BIP antibody, WB (1:1000), #3183, Cell Signaling Technology, MA
anti-cleaved-caspase 3 antibody, WB (1:500), #9664, Cell Signaling Technology, MA
anti-GAPDH antibody, WB (1:3000), #2118, Cell Signaling Technology, MA
anti-rabbit IgG HRP-linked antibody, WB (1:2000), NA934, GE Healthcare, UK
anti-mouse IgG HRP-linked antibody, WB (1:2000), NA931, GE Healthcare, UK
anti-goat-IgG biotin-conjugated antibody, IHC (1:500), AP180B, Millipore, MA
anti-rabbit-IgG biotin-conjugated antibody, IHC (1:500), AP182B, Millipore, MA

Validation

All of the antibodies used in this study are commercially available and validated for the application and species by the manufacturers, whose data are described at the manufacturer's website.

anti-ABCA1 antibody, NB400-105;

Reactivity; human, mouse, rat, porcine, canine, Chinese hamster, equine, hamster, primate, rabbit
Application; WB, ChIP, ELISA, Flow, IB, ICC/IF, IHC, IHC-Fr, IHC-P, IP, B/N, Dual ISH-IHC, Func, GS, KD, KO

anti-UCP1 antibody, U6382;
Reactivity; human, mouse, rat
Application; WB, IHC

anti-β-actin antibody, A5441;
Reactivity; human, mouse, rat, rabbit,pig, bovine, canine, feline, carp, guinea pig, sheep, Hirudo medicinalis
Application; WB, IHC, ELISA, IF

anti-tyrosine hydroxylase antibody, AB152;
Reactivity; human, mouse, rat, feline, ferret, squid, drosophila, mollusk
Application; WB, IHC, IHC-P, IF, ELISA, IP

anti-c-fos antibody, sc-52;
Reactivity; human, mouse, rat
Application; WB, IP, IF, IHC, flow cytometry, ELISA

anti-PGC1a antibody, H-300;
Reactivity; human, mouse, rat
Application; WB, IP, IF, ELISA

anti-CHOP antibody, sc-575;
Reactivity; human, mouse, rat
Application; WB, IP, IF, IHC, ELISA

anti-BIP antibody, #3183;
Reactivity; human, mouse, rat, monkey
Application; WB

anti-cleaved-caspase 3 antibody, #9664;
Reactivity; human, mouse, rat, monkey
Application; WB, IHC-P, IF-IC, Flow, IP

anti-GAPDH antibody, #2118;
Reactivity; human, mouse, rat, monkey, bovine, pig
Application; WB, IHC-P, IF-IC, Flow Cytometry

# Eukaryotic cell lines

Policy information about cell lines

| | |
|---|---|
| Cell line source(s) | Neuro2a cells were obtained from ATCC.<br>miR-33a knockout, miR-33b knockout, and miR-33a/b double knockout human iPS cells were generated using CRISPR-Cas9-mediated genome editing as reported previously (Nakazeki, et al. Clin Sci (Lond) 133, 583-595 (2019)). |
| Authentication | Cell lines were not authenticated in this study. |
| Mycoplasma contamination | Cell lines were tested negative for mycoplasma infection. |
| Commonly misidentified lines<br>(See ICLAC register) | No commonly misidentified cell lines were used. |

# Animals and other organisms

Policy information about studies involving animals; ARRIVE guidelines recommended for reporting animal research

| | |
|---|---|
| Laboratory animals | miR-33 knockout (miR-33-/-), miR-33b knock-in (miR-33b+/+), and miR-33flox (miR-33f/f) mice were originally generated in our laboratory as reported previously (Proc Natl Acad Sci U S A 107, 17321-6 (2010), Sci Rep 4, 5312 (2014), Circ Res 120, 835-847 (2017)). DBH-Cre mice were generated by Dr. Kazuto Kobayashi and provided by the RIKEN BRC through the National Bio-Resource Project of the MEXT, Japan (J Neurosci Res 78, 7-15 (2004)). All of the in vivo experiments were performed in C57BL/6J background mice, and littermates were used as controls. 8 week-old male mice were used in most experiments, except for oxygen consumption measurements of miR-33f / f DBH-Cre mice, in which 16 week-old male mice were used. All mice were maintained with free access to food and water in SPF condition. |
| Wild animals | This study did not involve wild animals. |
| Field-collected samples | This study did not involve field-collected samples. |

| Ethics oversight | All of the experimental protocols were approved by the Ethics Committee for Animal Experiments of Kyoto University. |
| --- | --- |

Note that full information on the approval of the study protocol must also be provided in the manuscript.
